# Nucleolin stabilizes G-quadruplex structures folded by the LTR promoter and silences HIV-1 viral transcription

**DOI:** 10.1093/nar/gkv897

**Published:** 2015-10-10

**Authors:** Elena Tosoni, Ilaria Frasson, Matteo Scalabrin, Rosalba Perrone, Elena Butovskaya, Matteo Nadai, Giorgio Palù, Dan Fabris, Sara N. Richter

**Affiliations:** 1 Department of Molecular Medicine, University of Padua, via Gabelli 63, 35121 Padua, Italy; 2 The RNA Institute, University at Albany-SUNY, Albany, NY 12222, USA

## Abstract

Folding of the LTR promoter into dynamic G-quadruplex conformations has been shown to suppress its transcriptional activity in HIV-1. Here we sought to identify the proteins that control the folding of this region of proviral genome by inducing/stabilizing G-quadruplex structures. The implementation of electrophorethic mobility shift assay and pull-down experiments coupled with mass spectrometric analysis revealed that the cellular protein nucleolin is able to specifically recognize G-quadruplex structures present in the LTR promoter. Nucleolin recognized with high affinity and specificity the majority, but not all the possible G-quadruplexes folded by this sequence. In addition, it displayed greater binding preference towards DNA than RNA G-quadruplexes, thus indicating two levels of selectivity based on the sequence and nature of the target. The interaction translated into stabilization of the LTR G-quadruplexes and increased promoter silencing activity; in contrast, disruption of nucleolin binding in cells by both siRNAs and a nucleolin binding aptamer greatly increased LTR promoter activity. These data indicate that nucleolin possesses a specific and regulated activity toward the HIV-1 LTR promoter, which is mediated by G-quadruplexes. These observations provide new essential insights into viral transcription and a possible low mutagenic target for antiretroviral therapy.

## INTRODUCTION

G-quadruplexes (G4s) are nucleic acids secondary structures that may form in single-stranded G-rich DNAs and RNAs under physiological conditions (1–3). Four Gs bind *via* Hoogsteen-type hydrogen bonds to yield G-quartets that in turn stack on top of each other to form the G4. G4s are highly polymorphic, both in terms of strand stoichiometry (forming both inter- and intramolecular structures) and strand orientation/topology. The presence of K^+^ cations specifically supports G4 formation and stability (4–6). In eukaryotes and prokaryotes, G4 DNA motifs have been found in telomeres, G-rich micro- and mini-satellites, near promoters, and within the ribosomal DNA (rDNA) (7–9). In the human genome, genes that are near G4 DNA motifs fall into specific functional classes; for example, promoters of oncogenes and tumor suppressor genes have particularly high and low G4-forming potential, respectively (10–12). Human G4 DNA motifs have been reported to be associated with recombination prone regions (13) and to show mutational patterns that preserved the potential to form G4 DNA structures (9). RNA G4s have been detected in the 5′ and 3′-UTR and coding regions, in which they act as important regulators of pre-mRNA processing (splicing and polyadenylation), RNA turnover, mRNA targeting and translation (14,15). Regulatory mechanisms controlled by G4s involve the binding of protein factors that modulate G4 conformation and/or serve as a bridge to recruit additional protein regulators. Indeed, G4 binding proteins can be classified into three functional groups: telomere-related proteins, such as the shelterin complex; proteins that unfold the G4 structure, such as the helicase and heterogeneous nuclear ribonucleoprotein families; proteins that stabilize G4s, a large group which includes nucleolin, MAZ and nucleophosmin (3,16–18).

G4 structures and their cognate proteins are key players in numerous essential processes in eukaryotic cells. Their misregulation has been associated with a number of relevant human diseases, such as the amyotrophic lateral sclerosis (19–21), Alzheimer (22) and fragile X syndrome (23), in which expansion of G4-forming regions has been reported. Moreover, mutations in G4-interacting proteins have been linked to genetic diseases, such as the Werner syndrome and Fanconi anemia (24,25). In recent years, new studies have contributed to increase our knowledge of the biological significance of G4s in prokaryotes (26,27) and viruses (28). We and other groups have identified functionally significant G4s in the Nef coding region (29) and the unique LTR promoter (30–32) of the human immunodeficiency virus (HIV), the etiologic agent of the acquired immune deficiency syndrome (AIDS). These studies have shown that G4 folding at the LTR promoter decreased viral transcription with an effect that was augmented by G4 ligands (30,33). In this direction, the significance of these structures as focal points of interactions with host and viral factors is supported also by the observation that G4-folded sequences are specifically recognized by various viral proteins, such as the Epstein Barr Virus Nuclear Antigen 1 (34,35) and the SARS coronavirus unique domain (SUD), which occurs exclusively in highly pathogenic strains (36). For this reason, we decided to pursue the investigation of putative cellular/viral proteins that may be involved in the regulation of the G4 LTR promoter activity in HIV. We employed a concerted approach combining electrophorethic mobility shift assay (EMSA) and analysis by electrospray ionization mass spectrometric (ESI-MS) to identify possible factors capable of binding the LTR G4 structure. In order to validate the findings, we then tested their stabilizing activity on the G4 fold and evaluated their ability to inhibit LTR-driven transcription in cells. The results provided new insights into the role of the LTR G4 in the viral life cycle, which could pave the way for the possible development of novel therapeutic strategies.

## MATERIALS AND METHODS

### Oligonucleotides, plasmids and aptamers

All desalted oligonucleotides and aptamers were purchased from Sigma-Aldrich, Milan, Italy (Supplementary Table S1). The HIV-1 LTR region was inserted into the promoterless luciferase reporter vector pGL4.10-Luc2 (Promega Italia, Milan, Italy) to form the pGL4.10-LTR-Luc2 vector, as previously reported (30). The Renilla plasmid (p4.74, Promega Italia, Milan, Italy) was used as an internal control. The human enhanced green fluorescent protein-nucleolin plasmid (GFP-Nucleolin) was purchased from Addgene (Addgene, Cambridge, MA, USA). The pEGFP empty vector was used as control (Clontech, Takara Bio, Otsu, Japan).

### Cell culturing

Human embryonic kidney (HEK) 293T cells (ATCC # CRL-3216) were grown in DMEM (Gibco, Thermo Fisher Scientific, Waltham, MA, USA) supplemented with 10% heat-inactivated fetal bovine serum (FBS, Gibco, Thermo Fisher Scientific, Waltham, MA, USA). Jurkat T-lymphocytes cells (ATCC # TIB-152) were grown in RPMI 1640 (Gibco, Thermo Fisher Scientific, Waltham, MA, USA) supplemented with 10% heat-inactivated FBS. MCF-7 human breast cancer cells (ATCC # HTB-22) were grown in RPMI 1640 supplemented with 10% heat-inactivated FBS. MCF10A normal human mammary epithelial cells (ATCC # CRL-10317) were grown in DMEM/F12 (Gibco, Thermo Fisher Scientific, Waltham, MA, USA) supplemented with 10% heat-inactivated FBS and EGF (0.02 μg/ml), hydrocortisone (0.5 μg/ml), cholera toxin (0.1 μg/ml), insulin (10 μg/ml) (purchased all from Sigma-Aldrich, Milan, Italy). All cultures were grown in a humidified incubator maintained at 37°C with 5% CO_2_.

### Protein nuclear extraction and electrophoretic mobility shift assay (EMSA)

Oligonucleotides were 5′-end labeled with [γ-^32^P]ATP using T4 polynucleotide kinase at 37°C for 30 min. After DNA precipitation, labeled species were resuspended in lithium cacodylate buffer (10 mM, pH 7.4) and KCl 100 mM. The oligonucleotides were denatured for 5 min at 95°C and gradually cooled to room temperature to achieve proper folding of G-quadruplex structures. Protein nuclear extracts of HEK 293T and Jurkat cells were obtained by using NXTRACT kit (Sigma-Aldrich, Milan, Italy). Recombinant full-length human nucleolin was expressed in HEK293 cells and purified as described by the manufacturer (Origene Technologies, Rockville, USA). Labeled oligonucleotides (40 nM) were incubated in 20 μl of reaction in EMSA binding buffer and nuclear extract (0.5 μg/μl) or purified nucleolin (300 ng) for 2 h at 37°C. EMSA binding buffer composition was: Tris–HCl 20 mM, pH 8, KCl 30 mM, MgCl_2_ 1.5 nM, DTT 1 mM, glycerol 8%, protease inhibitor cocktail (Sigma-Aldrich, Milan, Italy) 1%, NaF 5 mM, Na_3_VO_4_ 1 mM, poly [dI-dC] (Sigma-Aldrich, Milan, Italy) 1.25 ng/μl.

In competition experiments, an excess of cold oligonucleotides was added to the samples and their ability to disrupt the G4 structures was monitored to evaluate binding specificity. After incubation, reaction solutions were loaded onto 5% native polyacrylamide gel in 1× TBE buffer and KCl 100 mM. DNA–protein complexes were resolved by running the gel overnight at 27 V at 4°C. EMSA gels were dried using a gel dryer (Bio-Rad Laboratories, Milan, Italy), free and bound DNA molecules were visualized by phosphorimaging (Typhoon FLA9000, GE Healthcare Europe, Milan, Italy) and quantified by ImageQuant TL Software (GE Healthcare Europe, Milan, Italy). After the desired complex was located on the gel, the corresponding band was cut and either directly in-gel digested for mass spectrometric (MS) analysis, or further purified by SDS-PAGE. Extraction was performed in SDS-PAGE sample buffer. After 5 min in boiling water, samples were incubated overnight at 37°C. Finally, supernatant was loaded on 12% SDS-PAGE. The band of interest was excised after coomassie staining.

### Protein extraction from HIV-1 infected cells

HEK 293T cells were seeded in a 10-cm dish in DMEM supplemented with 10% heat-inactivated FBS and incubated overnight. Cells were next either mock-transfected or transfected with of pNL4-3 (the reagent was obtained throughout the NIH AIDS Reagent Program, Division of AIDS, NIAID, NIH) (37) using CalPhos™ Mammalian Transfection Kit (Clontech, Otsu, Japan) according to the manufacturer's protocol. After 8 h, cells were washed with PBS 1× and fresh growth medium was added. Forty eight hours post-transfection, cells were washed twice with cold PBS and scraped off. After a short centrifugation, pellet was resuspended in total protein extraction buffer (KCl 600 mM, Tris–HCl 20 mM, pH 7.8, glycerol 20%, DTT 2 mM, protease inhibitor cocktail). Cells were then lysed with three repeated freeze/thaw cycles and supernatant cleared by centrifugation, stored at −80°C, and subsequently used in EMSA assays.

### Mass spectrometric (MS) protein identification

Bands were treated according to established in-gel digestion protocols. Briefly, they were first washed with 50% CH_3_OH and 2.5% acetic acid, dehydrated with CH_3_CN, and then reduced with 30 μl of DTT (10 mM in 100 mM NH_4_HCO_3_) for 30 min at room temperature. The excess of DTT was eliminated before treating the bands with 30 μl of iodoacetamide (50 mM in 100 mM NH_4_HCO_3_) for 30 min at room temperature in order to alkylate cysteine residues. Bands were washed with 100 mM NH_4_HCO_3_, dehydrated with CH­_3_CN twice, and then digested. A 1 μg aliquot of MS-grade trypsin (ThermoFisher Scientific, Waltham, MA, USA) in 50 μl of 50 mM NH_4_HCO_3_ was added to the dehydrated bands, followed by incubation on ice for 30 min. The excess of trypsin was eliminated and substituted with 20 μl of 50 mM NH_4_HCO_3_ and the sample was incubated overnight at 37°C. Peptides were extracted twice with 5% formic acid and two more times with 50% CH_3_CN, 5% formic acid. The peptide mixture was further desalted in a silica nanocolumn (Polymicro Technologies, Phoenix, AZ, USA) packed in house with pinnacle C18 pack material (Thermo Fisher Scientific, Waltham, MA, USA). All materials were MS grade purchased from Sigma Aldrich, St. Louis, MO, US except where otherwise indicated.

The desalted mixture was finally analyzed by direct infusion electrospray ionization (ESI) on a Thermo Fisher Scientific (Waltham, MA, USA) LTQ-Orbitrap Velos mass spectrometer. The instrument was calibrated by using a 0.5 mg/ml solution of CsI in 50% CH_3_OH, which provided a typical <2 ppm mass accuracy. All analyses were performed in nanoflow mode by utilizing quartz emitters produced in house by using a P2000 laser pipette puller (Sutter Instruments Co., Novato, CA, USA). Up to 5 μl samples were typically loaded onto each emitter by using a gel-loader pipette tip. A stainless steel wire was inserted through the back-end of the emitter to supply an ionizing voltage that ranged between 0.8 and 1.2 kV. Bands containing bovine serum albumin (BSA) and empty gel bands were used as positive and negative control, respectively. Putative peptides that were not present in blank samples were submitted to tandem mass spectrometric (MS/MS) analysis. These determinations involved isolating the precursor ion of interest in the LTQ element of the instrument, activating fragmentation in either the LTQ or the C-trap, and performing fragment detection in the Orbitrap. The masses of the 50 more intense fragments were employed to perform a Mascot Database Search (38) to identify their parent protein. The matched protein was deemed as being positively identified when two or more peptides provided a mascot score greater than 22.

### Pull-down assay

HEK 293T protein nuclear extract (0.6 μg/μl) was incubated with biotinylated LTR oligo G4 folded (600 nM) in 250 μl of reaction containing Tris–HCl 20 mM, pH 8, KCl 30 mM, MgCl_2_ 1.5 nM, protease inhibitor cocktail 1%, NaF 5 mM, Na_3_VO_4_ 1 mM, poly [dI-dC] 1.25 ng/μl for 2 h at 37°C. The binding reaction was followed by incubation (2 h at 37°C) with 30 μl of streptavidin-agarose beads (Sigma-Aldrich, Milan, Italy). After PBS washes, proteins were eluted with increasing amount of NaCl (0.2 and 1M), and concentrated with Amicon Ultra 0.5 (Merck Millipore, Germany). Beads were collected by brief centrifugation, resuspended in 50 μl of Laemmli buffer, and finally incubated at 95°C for 5 min. Supernatants were separated on SDS-PAGE and analyzed by western blot.

### FRET-melting assay

Oligonucleotides were diluted to 0.1 μM in lithium cacodylate buffer (10 mM, pH 7.4) and KCl 100 mM heat denatured for 5 min at 95°C, and folded in G4 structure at room temperature for 16 h. Samples were incubated alone, with purified human nucleolin (530 ng) or bovine serum albumin (BSA, negative control) for 1h at 37°C. Fluorescence melting curves were determined by using a LightCycler II (Roche, Milan, Italy). After a first equilibration step at 30°C for 2 min, a stepwise increase of 1°C every minute for 65 cycles was performed to reach 95°C. A measurement was completed after each cycle by using 470 nm excitation and 530 nm detection. Oligonucleotide melting was monitored by observing 6-carboxyfluorescein (6-FAM) emission, which was normalized between 0 and 1. *T*_m_ was defined as the temperature for which the normalized emission was 0.5.

### siRNA and luciferase reporter assay

Gene-specific pooled siRNA trilencer targeting human NCL and a scrambled negative control duplex were purchased from Origene (NCL Trilencer-27 Human siRNA, OriGene Technologies, Rockville, MD, USA). MCF7 cells were transfected with 1, 2 and 4 nM aliquots of human NCL siRNA and control siRNA by using Lipofectamine RNAiMAX (Invitrogen, Thermo Fisher Scientific, Waltham, MA, USA) following the manufacturer's instructions. pLTR luciferase plasmid and Renilla construct were transfected into the same cells 24 h later by using Lipofectamine 3000 (Invitrogen, Thermo Fisher Scientific, Waltham, MA, USA). In the double transfected cells, LTR promoter activity was assessed as firefly luciferase signal, normalized to Renilla luciferase activity, by using Dual-Glo^®^ Luciferase Assay System (Promega Italia, Milan, Italy), according to the manufacturer's directions (30). Depending on the transfected cell line (i.e. either MCF7 or MCF10A), the pGL4.10-LTR-Luc2 vector provided signals ranging form 10^3^ to 2 × 10^6^ luciferase units, as measured by a Victor X2 multilabel plate reader (Perkin Elmer Italia, Milan, Italy). In contrast, the promoterless pGL4.10-Luc2 and untransfected cells displayed a background signal lower than 10 luciferase units. All data were acquired in medium-free PBS. DNA aptamers AS1411 and the control CRO26 were added to cell medium at the time of transfection of pGL4.10-LTR-Luc2 and p4.74-Renilla plasmids and the luciferase signal read 24 h after transfection. Each assay was performed in duplicate and each set of experiments was repeated at least three times.

### Immunoblot analysis

Immunoblot analysis was performed on cell protein extracts obtained as previously described (39). Protein concentrations were quantified by using the Pierce^®^ BCA Protein Assay Kit (Thermo Scientific, Rockford, IL, USA) and the samples stored at −80°C. Each sample was electrophoresed on 12% SDS-PAGE and transferred to a nitrocellulose blotting membrane (Amersham TM Protan TM, GE Healtcare Life science, Milan, Italy) by using trans-blot SD semi-dry transfer cell (Bio-Rad Laboratories, Milan, Italy). The membranes were blocked with 5% skim milk in PBST (0.05% Tween 20 in PBS). Membranes were incubated with the respective primary antibody directed against NCL (rabbit polyclonal C23 (H-250); Santa Cruz Biotechnology, Dallas, TX, USA), p24 (rabbit polyclonal; Abcam, Cambridge, UK), and β-actin (mouse monoclonal; Sigma-Aldrich, Milan, Italy). After three washes in PBST, membranes were incubated with ECL Plex Goat-α-Rabbit IgG-Cy5 or ECL Plex Goat-α-Mouse IgG-Cy5 (GE Healthcare Life sciences, Milan, Italy). Images were captured on the Typhoon FLA 9000, and quantified by ImageQuant TL software.

### Taq polymerase stop assay

Taq polymerase stop assay was carried out as previously described (30). Briefly, the 5′-end labeled primer (5′-GGCAAAAAGCAGCTGCTTATATGCAG-3′) was annealed to the template (Supplementary Table S1) in lithium cacodylate buffer in the presence or absence of KCl 100 mM by heating at 95°C for 5 min and gradually cooling to room temperature. Where specified, samples were incubated with purified human nucleolin (61 ng) at 37°C for 2 h. Primer extension was then conducted by using 2 U of Ampli*Taq* Gold DNA polymerase (Life Technologies, Thermo Fisher Scientific) for 30 min at 37°C or 47°C. Reactions were stopped by ethanol precipitation. Primer extension products were separated on a 15% denaturing gel, and finally visualized by phosphorimaging (Typhoon FLA 9000).

### Dimethylsulfate footprinting

The DNA substrate of interest was gel-purified before use and prepared in desalted/lyophilised form. The oligonucleotide was 5′-end-labeled with [γ-^32^P]ATP by T4 polynucleotide kinase, purified by using MicroSpin G-25 columns (Amersham Biosciences, Europe), resuspended in lithium cacodylate buffer 10 mM, pH 7.4, KCl 100 mM, heat-denatured and folded. The oligonucleotide (1.23 μM) was incubated either alone or with purified human nucleolin (300 ng) in EMSA binding buffer for 2 h at 37°C. Sample solutions were then treated with dimethylsulfate (DMS, 0.5% in ethanol) for 5 min and stopped by addition of gel loading buffer containing 10% glycerol and β-mercaptoethanol. Samples were loaded onto 16% native polyacrylamide gels and run until the desired resolution was obtained. DNA bands were localized via autoradiography, excised and eluted overnight. The supernatants were recovered, ethanol-precipitated and treated with piperidine 1M for 30 min at 90°C. Samples were dried in a speed-vac, washed with water, dried again and resuspended in formamide gel loading buffer. Reaction products were analyzed on 20% denaturing polyacrylamide gels, visualized by phosphorimaging analysis, and quantified by ImageQuant TL software.

### Surface plasmon resonance (SPR) analysis

SPR was performed on the Biacore T100 platform (GE Healthcare, Life Science, Milan, Italy). Purified human NCL was immobilized on Serie S sensor chip CM5 by amine coupling. Immobilization was performed in HEPES–NaCl running buffer (HEPES pH 7.4 0.01 M, NaCl 0.15 M, EDTA 3 mM). The protein diluted in sodium acetate buffer pH 4.0 at the concentration of 15 ng/μl was injected to reach the response of around 1000 RU. Blank immobilization was performed in the flow cell 1 to permit reference subtraction. LTR-II+III+IV wt and scrambled sequences binding analysis was performed at a flow rate of 25 μl/min, with contact time of 180 s and dissociation time of 240 s in HEPES-KCl buffer (HEPES pH 7.4 15 mM, KCl 0.25 M, EDTA 3 mM). Oligonucleotides were diluted from stock to the concentration of 2 μM in HEPES-KCl buffer, denaturated at 95°C for 5 min and allowed to cool at room temperature to permit G4 formation. Sensograms were obtained in the concentration range of 31.25 nM–2 μM. After each oligonucleotide injection the chip surface was regenerated with KCl 1M solution. All sensograms were corrected by reference subtraction of blank flow cell response and buffer injection response. Data were fitted to a 1:1 binding model with Rmax initial parameter set to theoretical calculated Rmax of 157 using BIAevaluation software (GE Healthcare).

## RESULTS

### A cellular protein binds specific G-quadruplexes of the HIV-1 LTR promoter

**Figure 1.**
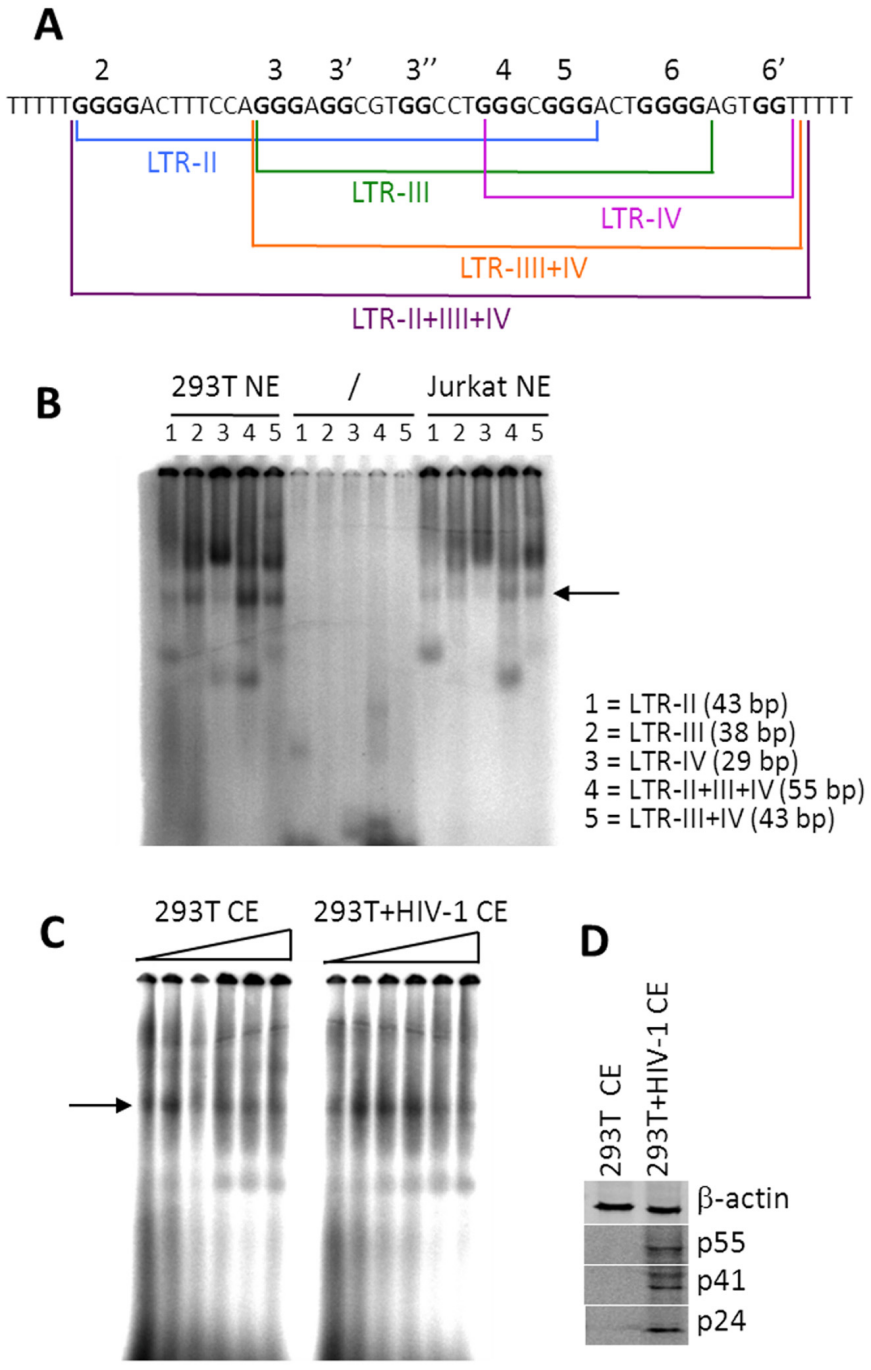
EMSA analysis of cellular and viral proteins binding to the G4 LTR sequences. (**A**) Sequence of the G-rich LTR region spanning −92/−48 nts. G bases in G-tracts or involved in G-quadruplexes are shown in bold. G-tracts are numbered. Brackets indicate sequences used in this study that form G-quadruplexes with three stacked tetrads. (**B**) Analysis of cellular proteins that bind to the LTR sequences. Protein nuclear extracts (NE) of 293T and Jurkat cells were incubated with the different length G4 LTR sequences. Binding was analysed by native polyacrylamide gel. Lane numbers correspond to the different LTR sequences, as shown in the legend at the right bottom of the gel image. Length of the LTR oligonucleotides is shown in brackets (see also Supplementary Table S1 for reference). The relevant protein/DNA complex band present in all G4 LTR sequences is indicated by the arrow. (**C**) Analysis of viral proteins that bind to the LTR-II+III+IV sequence. Cell extracts (CE) of 293T cells either non-producing or producing HIV-1 were incubated with the LTR-II+III+IV G4 and analysed by native polyacrylamide gel. The relevant protein/DNA complex band present in all G4 LTR sequences is indicated by the arrow. D) Western blot analysis of cell extracts (CE) of 293T cells either non-producing or producing HIV-1. The effective presence of the virus in transfected cells was evaluated with a HIV-1 p24 antibody.

In previous work, we demonstrated that the HIV-1 LTR region can fold at least three different G-quadruplex (G4) structures at positions −92/−48 with respect to the transcription initiation site of the representative HXB2_LAI (NC_001802) strain (30). Disruption of G4 by point mutations increased the transcript levels, thus indicating that G4 formation may contribute to the modulation of viral transcription. We reasoned that stabilization and unfolding of G4 at the LTR level are likely regulated by interactions with viral/cellular proteins. In order to test this hypothesis, LTR G4-forming sequences were incubated with nuclear protein extracts. LTR sequences of different lengths were employed to check whether individual G4 structures identified in the LTR promoter displayed different protein binding capabilities. In particular, we assayed sequences that possessed the minimal requirements to fold into an individual G-quadruplex (i.e. only 4 G-tracts: LTR-II, LTR-III and LTR-IV) and sequences capable of providing multiple G4s (i.e. more than 4 G-tracts: LTR-II+III+IV and LTR-III+IV) (Figure 1A). The corresponding ^32^P-labeled oligonucleotides were incubated with nuclear extracts derived from two cell lines: Jurkat T-lymphocytes that are a model for the natural HIV-1 targets *in vivo*; human embryonic kidney 293T cells that lack HIV-1 cell receptors and sustain all viral steps with the exception of virion attachment and entry. The latter can however be transfected with the HIV-1 proviral genome to produce fully competent and infectious viral particles, which indicates that their cytoplasmic/nuclear protein makeup is competent to sustain viral replication. Samples were analysed onto native polyacrylamide gels to monitor the formation of slower running bands corresponding to oligonucleotide-protein complexes. As shown in Figure 1B, all G4 LTR oligonucleotides formed slower running bands. The fact that the patterns obtained from the two nuclear extracts were essentially identical indicated that the selected cell lines contained very similar sets of G4-binding proteins. In particular, a very specific band migrated with the same rate in all G4 oligonucleotide samples (arrow in Figure 1B), thus suggesting that the same protein was able to bind all LTR G4 sequences considered. The intensity of the observed bands indicated that the longer sequence (i.e. LTR-II+III+IV) was bound most efficiently, whereas the shorter sequence (i.e. LTR-IV) supported only very modest complex formation. The LTR-II+III+IV oligonucleotide was incubated with extracts of HIV-1 producing and non-producing 293T cells to test whether the presence of viral proteins affected in any detectable way the observed EMSA profiles (Figure 1C). The actual presence of viral proteins in the transfected cells was assessed by western blot analysis (Figure 1D). While viral proteins were well represented in the transfected extract, the EMSA determinations revealed no major difference and confirmed that cellular proteins must constitute the major players in LTR G4 binding (Figure 1C).

**Figure 2.**
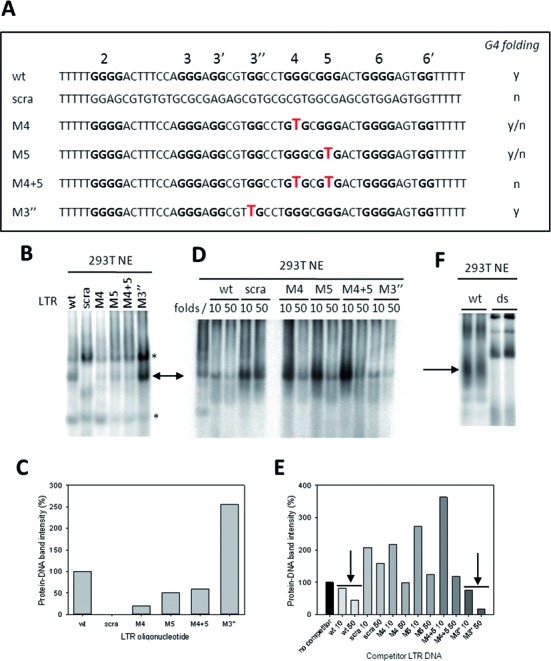
Binding of nuclear extract proteins to the wild-type (wt) and mutant LTR-II+III+IV sequences. (**A**) Sequences of the wt and mutant LTRs. G-tracts are shown in bold and are numbered (2–6’) above the sequence. In the mutant sequences, the mutated bases are shown in red. The names of the mutant sequences correspond to the G-tracts where G bases have been mutated. Scra stands for a sequence where Gs have been scrambled to such an extent that no G4 can form. The ability of the mutant sequences to fold into G4 is reported on the right: y = folding, y/n = partial folding, n = no folding (measured as reported in (30)). (**B**) Binding of nuclear extract proteins to the wt and mutant labeled G4 LTR sequences. The G4-specific protein/DNA complex is indicated by the arrow, while the asterisks indicate non-specific protein/DNA complexes. (**C**) Quantification of G4-specific protein/DNA complex reported in (B). (**D**) Competition of the G4-specific protein/DNA complex with unlabelled wt and mutant LTR sequences. The molar excess of the competitor sequences is shown above each lane. (**E**) Quantification of G4-specific protein/DNA complex reported in (D). The arrows indicate the only two unlabeled sequences able to compete the wt LTR/protein complex. (**F**) Binding of different amounts of nuclear extract proteins to the wt G4 folded and ds LTR-II+III-IV sequences.

In previous work, we demonstrated that one- or two-nucleotide point mutations in the G-tracts involved in G4 pairing were able to partially or totally disrupt, respectively, G4 folding in the LTR sequence (LTR-M4, LTR-M5, LTR-M4+5), whereas a mutation in the loop was not (LTR-M3’’, Figure 2A) (30). Based on these observations, we used mutant LTR-II+III+IV oligonucleotides to assess the specificity of the observed protein for the G4 structure (Figure 2B). When incubated with the nuclear extracts, only the sequences that retained G4-folding capabilities (i.e. wt and M3’’) were able to form bands corresponding to the desired complexes (arrow in Figure 2B), whereas the mutants that partially fold or cannot form stable G4s were much less efficiently bound. A scrambled sequence matching the wt base composition did not bind at all (Figure 2B and C). It should be noted that both a slower and a faster running bands were present in all samples (see asterisk in Figure 2B), indicative of nucleic acid binding proteins that may not be selective for G4. A competition experiment was performed to confirm the binding specificity of the selected protein, which involved mixing the labeled wt sequence with increasing amounts of unlabeled wt, mutant LTRs or scrambled sequence. As shown in Figure 2D and E, only the wt and M3’’ sequences were able to effectively compete for protein binding, whereas the other oligonucleotides did not decrease the amount of protein bound to wt. Interestingly, however, 10-fold addition of M4, M5 and M4+5 oligonucleotides increased the amount of protein bound to the wt sequence. In this case, the mutant sequences may compete for binding to proteins that are sequence- but not structure-specific, thus leaving more G4-specific protein available to bind to the G4-folded wt sequence. Furthermore, the fact that wt sequences folding G4 or ds structures bound different proteins confirmed that the observed protein was specific for the G4 conformation (Figure 2F).

### Nucleolin specifically recognizes and stabilizes the LTR G-quadruplexes

**Figure 3.**
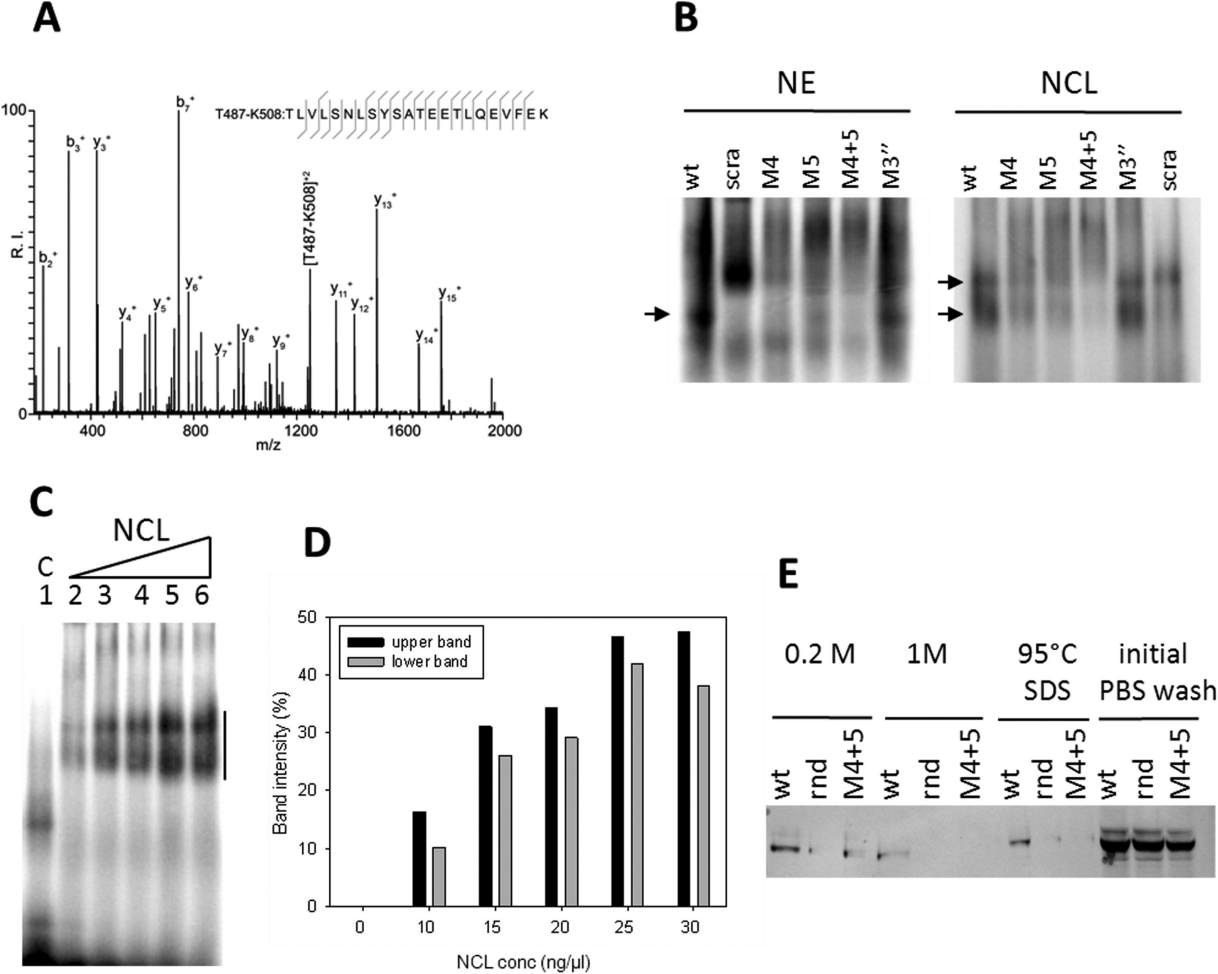
Identification of the G4-specific LTR binding protein. (**A**) MS/MS spectrum of the precursor ion observed at *m*/*z* 1251.14 in the sample mixture from native gel 1 (see Table 1). Only characteristic b and y ions are indicated (40,41). The data matched the sequence of peptide T487-K508 of NCL, which is reported on top with the observed fragments. (**B**) EMSA analysis of the binding of nuclear extract (NE) proteins and purified NCL to the wt and mutant LTR sequences. Arrows indicate relevant protein/LTR G4 complex bands. (**C**) EMSA analysis of the binding of increasing amounts of purified NCL to the wt LTR-II+III+IV G4. The vertical bar highlights the portion of the gel where the two NCL/LTR G4 complex bands are observed. (**D**) Quantification of the upper and lower NCL/LTR G4 complex bands obtained in the EMSA in panel (C). (**E**) Pull-down assay of nuclear extract proteins with wt, mutant G4 LTR-II+III+IV and random (rnd) sequences, immobilized on agarose beads. Shown is the western blot analysis with an NCL antibody. Proteins complexed to the beads-bound LTRs were washed with augmented stringency by increasing the ionic strength of the wash buffer (0.2 and 1 M). The final elution was obtained in denaturing buffer at 95°C.

With the goal of identifying the protein of interest, the relevant EMSA band was excised from the gel and submitted directly to trypsin digestion (see Materials and Methods). Alternatively, the material extracted from the EMSA band, which could possibly include multiple co-migrating species, was further purified on a SDS gel before trypsin digestion. In either case, the digestion products were subjected to MS/MS analysis and database searching (Figure 3A) (40,41), which provided an excellent match with human nucleolin (NCL) (Table 1). The experiment was separately repeated three times to corroborate the results. Positive identification was also confirmed by performing EMSA analysis of samples that included the G4-folded wt and mutant LTR-II+III+IV sequences with either nuclear extracts or purified human NCL. NCL displayed the same binding activity towards the wt and mutant LTR sequences manifested by the unknown protein (Figure 3B). NCL binding to the wt LTR-II+III+IV G4 was concentration dependent (Figure 3C and D). Surprisingly, however, two NCL/LTR complex bands were identified (Figure 3B and C). It has been reported that both native and purified NCL display self-cleaving activity and indeed preparations of NCL usually exhibit multiple bands (42). Our purified NCL also showed multiple bands on SDS gel, as detected by both Coomassie staining and western blot analysis (Supplementary Figure S1A). MS analysis of the four bands detected by Coomassie staining showed full-length peptide coverage only for the upper major band, whereas coverage of the N-terminal portion was missing in the lower three bands (Supplementary Figure S1B). We therefore ascribed the upper and lower bands to the full-length and cleaved forms of NCL, respectively. Interestingly, the band obtained from nuclear extracts migrated the fastest and, indeed, the N-terminal portion was not observed by MS peptide analysis (Table 1 and Supplementary Figure S1B). In addition, when the protein bound to LTR G4 in the EMSA gel was extracted and analyzed separately on SDS gel, it migrated at around 75 kDa, corresponding to one of the cleaved forms of NCL (data not shown). Therefore, we conclude that the NCL form present in the nuclear extracts corresponds to its cleaved portion lacking the N-terminus. In addition, NCL formed a complex with a scrambled sequence, which displayed a slightly slower migration rate compared to the G4-bound NCL (Figure 3B). The amount of complex was similar to that afforded by M4 and M5 LTR sequences. Binding of NCL to G-rich oligonucleotides has been previously reported (43) and provides an excellent indication that the RNA binding domains (RBD) of the purified NCL are indeed active. In analogous fashion, biotinylated wt, M4+5 LTR-II+III+IV, and scrambled-sequence oligonucleotides were incubated with nuclear extracts and then added to streptavidin-functionalized agarose beads to facilitate removal of unbound proteins. Immobilized proteins were eluted with buffers of increasing ionic strength and identified by western blot analysis (Figure 3E). NCL was released from the wt sequence when treated with increasing concentrations of NaCl or heated to 95°C in denaturing buffer, whereas it was released from the M4+5 and scrambled sequences at the lowest NaCl concentration, consistent with a lower affinity for these non-G4-forming sequences. These data confirm that NCL specifically binds the G4 folded conformation of the LTR-II+III+IV sequence.

**Table 1.** MS analysis of protein content of three gel bands collected from one SDS gel and two native gels

Sample	Experimental monoisotopic mass	Expected monoisotopic mass	Protein match	Peptide match	Score
SDS gel	2199.02	2199.02	Nucleolin	(G578-R597)	22
	1647.73	1647.73	Nucleolin	(F349-K362)	63
	1560.67	1560.67	Nucleolin	(G611-K624)	68
					
Native gel 1	4856.25	4856.26	Translationally-controlled tumor protein	(T39-K85)	22
	2500.26	2500.26	Nucleolin	(T487-K508)	102
	2311.14	2311.15	Nucleolin	(V298-K318)	84
	1647.73	1647.73	Nucleolin	(F349-K362)	71
					
Native gel 2	2500.26	2500.26	Nucleolin	(T487-K508)	93
	2311.14	2311.15	Nucleolin	(V298-K318)	62
	2199.02	2199.02	Nucleolin	(G578-R597)	30
	1560.67	1560.67	Nucleolin	(G611-K624)	60

**Figure 4.**
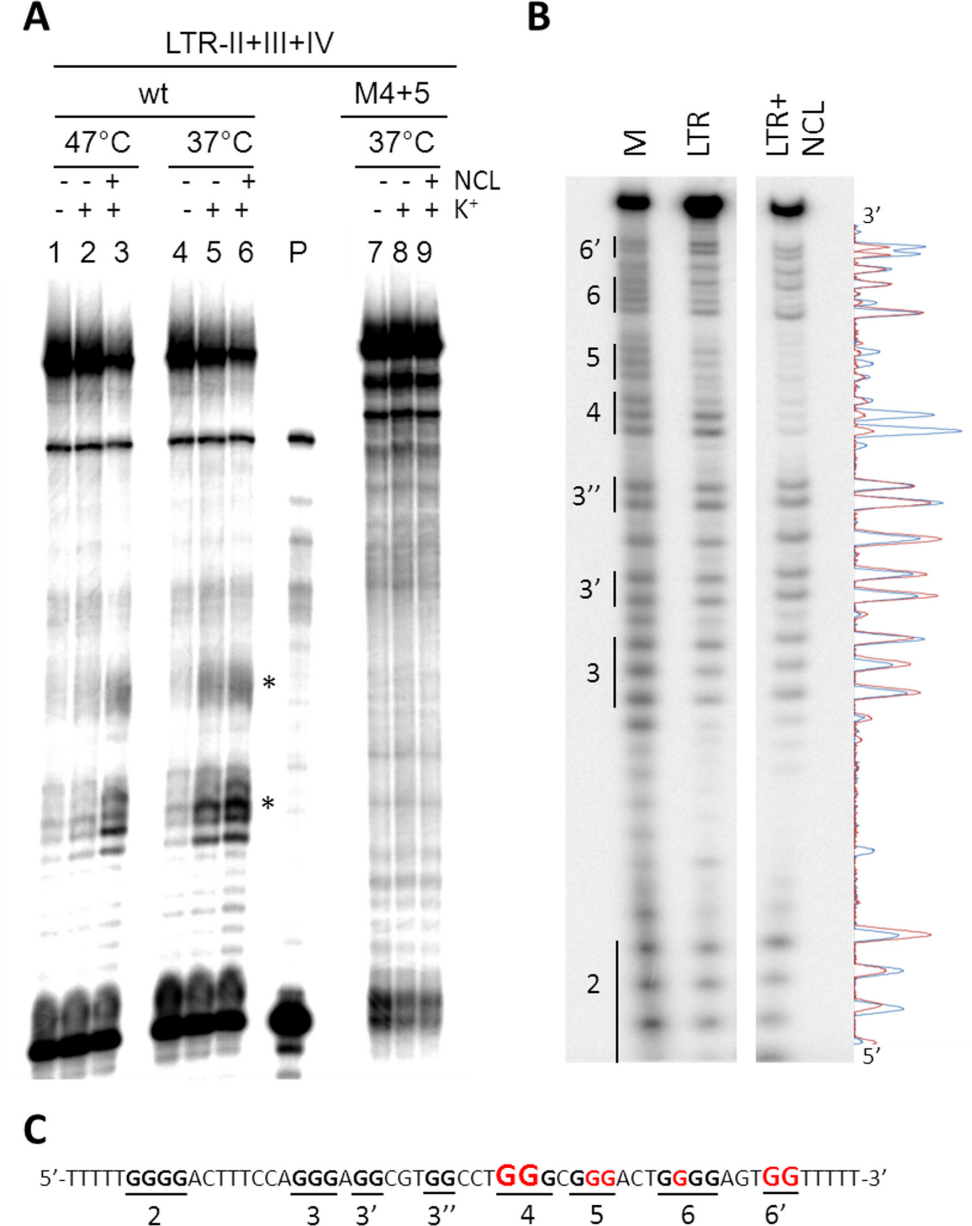
Characterization of NCL binding to the G4 LTR-II+III+IV. (**A**) Taq polymerase stop assay. Taq polymerization was performed in the presence/absence of K^+^ and NCL, as indicated, on the wt and M4+5 LTR-II+III+IV sequences. Amplification of the wt template was performed at 37°C and 47°C; on the mutant template elongation was obtained at 37°C. Stop regions are highlighted by the * symbol. (**B**) DMS protection analysis of the NCL/LTR complex and the G4 LTR-II+III+IV oligonucleotide. Band cleavage intensity is quantified by a densitogram shown on the right: red and blue lines correspond to the LTR and NCL/LTR complex, respectively. G-tracts and their numbering are indicated on the left. The two gel portions derive from a single gel run. (**C**) The LTR-II+III+IV sequence: bases protected by the DMS alkylation/cleavage are shown in red. The font of the red letters is proportional to the protection. G-tracts are numbered.

Taq polymerase stop assays were performed to assess the stabilization imparted by NCL to the various LTR G4 structures. The wt LTR-II+III+IV and M4+5 mutant sequences were extended to include a primer-annealing region and used as templates for a single-cycle Taq reaction (Supplementary Table S1). Elongation of the wt template was performed at 37°C or 47°C in the presence/absence of NCL. In samples containing 100 mM KCl and no NCL, a pausing site corresponding to the most 3′-end G-tract was observed only in the reaction elongated at 37°C. The pause was not observed at 47°C, consistent with possible destabilizing effects of temperature on the G4 structure (compare lanes 2 and 5 of Figure 4A). Addition of NCL induced more evident pauses at both elongation temperatures (Figure 4A, lanes 3 and 6), which clearly highlighted the stabilizing properties of NCL on G4 conformation. As expected from the inability of the M4+5 mutant to fold G4 structures, no pausing sites were observed regardless the presence of protein (Figure 4A, lanes 7–9), thus confirming the specificity of NCL binding for full-fledged G4s. At the same time, FRET melting assays were also carried out to study the stabilizing effects of NCL on the G4 structures. The assays involved the synthesis of constructs that combined selected G4-forming sequences, such as LTR-II+III+IV and the shorter LTR-II, LTR-III, LTR-IV, and LTR-III+IV (Figure 2A), with *FAM* and *TAMRA* moieties placed at their 5′- and 3′-ends, respectively. The results showed that NCL conferred the highest stabilization in the series to the LTR-II+III+IV construct, followed by LTR-III+IV. Progressively lower stabilization was observed for LTR-III and LTR-II, whereas LTR-IV was the least affected in the series (Table 2). The negative control bovine serum albumin (BSA) did not afford any detectable stabilization to the selected sequences.

**Table 2.** FRET analysis of the stabilization of NCL on the different length G4 LTR sequences

LTR sequence	*T*_m_ LTR (°C)	*T*_m_ LTR + NCL (°C)	Δ*T*_m_ (°C)
LTR-II	58.1 ± 0.2	76.2 ± 1.4	18.1
LTR-III	63.1 ± 0.1	75.1 ± 0.7	12.0
LTR-IV	62.1 ± 0.6	69.0 ± 1.4	6.9
LTR-III+IV	54.0 ± 0.6	78.5 ± 2.5	24.5
LTR-II+III+IV	48.0 ± 0.6	86.9 ± 1.4	38.9

To identify the position of putative NCL binding sites onto the DNA G4 structures, we performed dimethylsulfate (DMS) footprinting. When a complex of NCL with the LTR-II+III+IV DNA was assessed (Figure 4B), the methylation pattern revealed that the 3′ region of the LTR sequence was protected by protein binding with unique specificity for two G bases in G-tract 4, as highlighted in Figure 4C.

**Figure 5.**
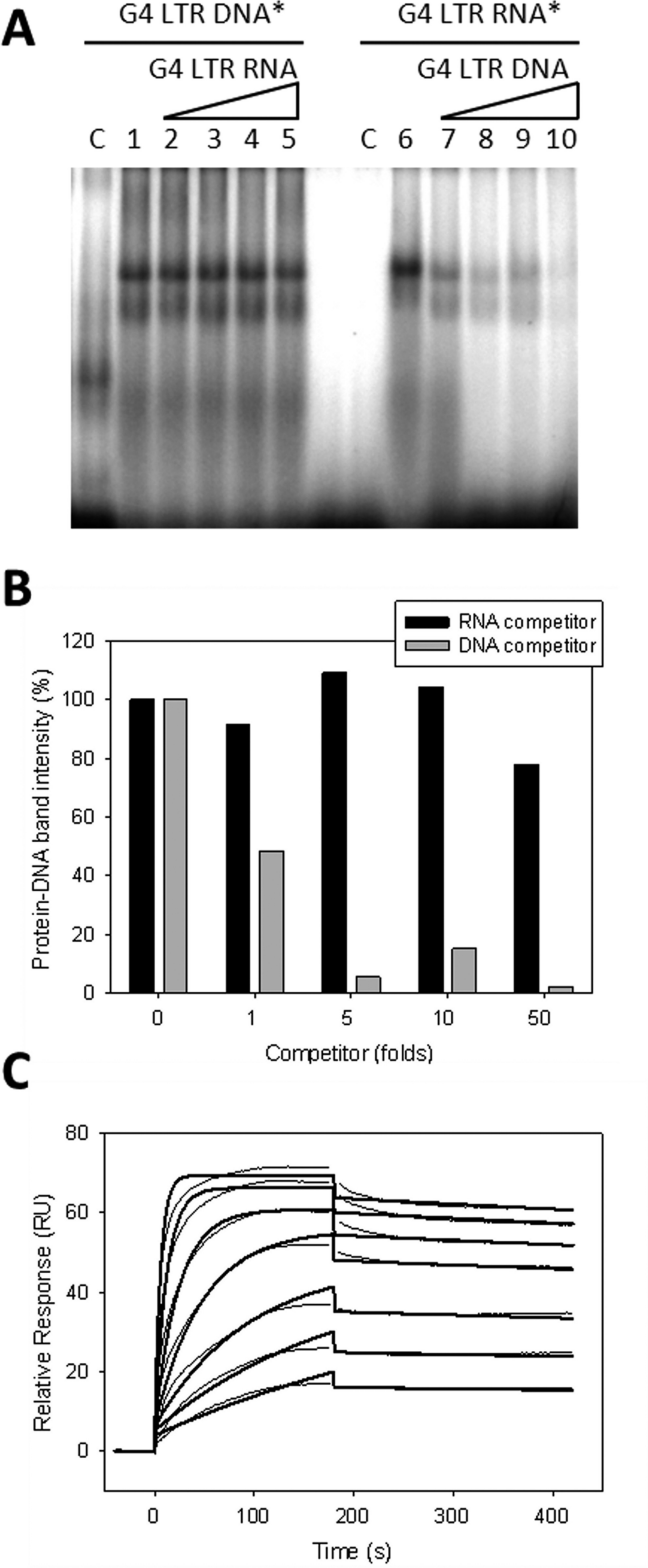
Properties of NCL binding to the G4 LTR-II+III+IV sequence. (**A**) EMSA competition analysis of NCL binding towards the G4 LTR-II+III+IV DNA and RNA sequences. On the left: the labeled G4 LTR DNA* was bound to NCL and competed with increasing folds of unlabelled G4 LTR RNA (1–50 folds); on the right, the labeled G4 LTR RNA* was bound to NCL and competed with increasing folds of unlabelled G4 LTR DNA (1–50 folds). C is a control lane for the oligonucleotides before binding. (**B**) NCL/LTR complex band quantification of the experiment presented in (A). (**C**) SPR binding analysis of wt LTR-II+III+IV to immobilized NCL. Oligonucleotide concentration range was 31.25 nM-2000 nM. Sensograms are shown as gray lines and their respective fits as black lines.

Based on the facts that NCL has been described as a RNA-binding protein (44) and that the LTR sequence is present in the U3 region of the HIV-1 genome during the initial infection steps, we tested whether NCL could also bind the RNA version of the LTR G4. In this case, NCL was incubated with labeled RNA or DNA oligonucleotides capable of folding LTR G4s. Increasing concentrations of unlabelled RNA and DNA counterparts were employed to compete for NCL binding. The results clearly showed that the protein was able to bind both types of G4 oligonucleotides (lanes 1 and 6, Figure 5A). However, the DNA G4 was consistently able to outcompete the RNA version for protein binding (lanes 7–10, Figure 5A and B), whereas the RNA was incapable of outcompeting the DNA (lanes 2–5, Figure 5A and B). We had previously shown that the LTR RNA G4s fold into parallel structures (33), whereas the DNA counterparts display rather hybrid-like conformations (30); therefore, the preferential binding toward the DNA G4 is likely caused by these substantial structural differences between the DNA and RNA G4 conformations.

Surface plasmon resonance (SPR) analysis was next used to assess the affinity of the purified NCL for the wt LTR-II+III+IV G4: a *K*_D_ of 2.5 ± 0.1 nM was obtained, which indicates an extremely high affinity of the protein for this LTR G4 (Figure 5C). In contrast, the scrambled oligonucleotide showed no affinity for NCL (Supplementary Figure S1).

### Binding of nucleolin to the LTR G-quadruplexes increases repression of viral transcription

**Figure 6.**
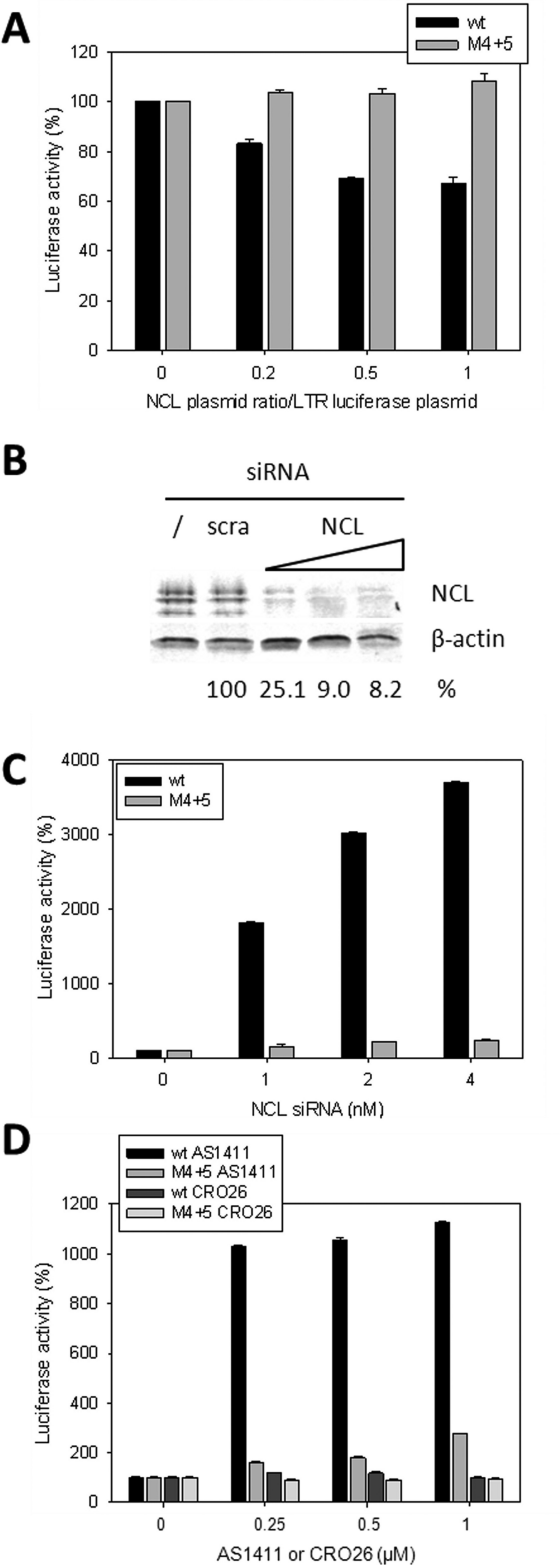
Activity of NCL on the HIV-1 LTR promoter in cells. (**A**) Analysis of the luciferase activity of the wt and M4+5 LTR promoters in MCF-10A cells transiently transfected with the LTR luciferase plasmids and a NCL expression plasmid. (**B**) NCL depletion in MCF7 cells by NCL siRNAs analysed by western blot with NCL antibody. Scra indicates scrambled siRNAs. Detection of β-actin was used as control. The % of NCL expression in each sample is given below the gel. (**C**) Analysis of the luciferase activity of the wt and M4+5 LTR promoters in MCF7 cells treated with NCL siRNAs. (**D**) Analysis of the luciferase activity of the wt and M4+5 LTR promoters in MCF7 cells treated with the NCL-binding aptamer AS1411 and the negative control CRO26.

A luciferase reporter assay was established to explore the downstream biological effects of NCL binding to the LTR promoter. Two epithelial breast cell lines were selected for the assay: MCF-7 breast cancer cells and MCF-10A normal breast epithelial cells. The latter inherently express lower amounts of NCL compared to tumor cells (45,46). For this reason, the MCF-10A cells were transfected with wt or M4+5 LTR luciferase reporter plasmids, either alone or in the presence of increasing amounts of NCL expression vector. The luciferase signal was measured to determine the level of activation of the LTR promoter. The results showed that the activity of wt LTR decreased to 65% of the control, while that of the M4+5 promoter remained unvaried (Figure 6A). The MCF7 cell line that overexpresses NCL was employed to perform additional activity assays. In this case, cells were treated with increasing amounts of siRNAs designed to target NCL mRNA (47), and then transfected with wt or mutant M4+5 LTR luciferase reporter plasmids. Analysis of NCL content showed that the protein was effectively depleted at 1–4 nM siRNA, reaching 8% of the initial amount at 4 nM (Figure 6B). Measured by luciferase activity, the effect of NCL depletion on LTR promoter activity was quite astonishing: at 4 nM of siRNA, the promoter activity of the wt sequence was 37 folds that of the control while the mutant M4+5 sequence was only 1.5 folds (Figure 6C). As a complementary approach, we tested the effect of the NCL-targeted DNA aptamer AS1411, which has been reported to bind with high affinity NCL in cells (48). At 1 μM of AS1411, we again observed a significant increment of the wt LTR promoter activity to reach 11 times that of the non-treated control while the mutant M4+5 sequence increased of only 2.5 times (Figure 6D). In contrast, the control sequence CRO26 that is complementary to AS1411 did not modify LTR promoter activity. These data indicate that the specific binding of NCL to G4 structures in the LTR promoter exerts significant repressive effects on HIV-1 promoter activity.

## DISCUSSION

We have identified NCL as a prominent host factor capable of binding with high affinity to the G4 structures present in the LTR promoter of HIV-1. We observed that the specific interaction leads to G4 stabilization and contributes to silence viral transcription. Conversely, we also demonstrated that NCL depletion produces extraordinary enhancing effects on LTR promoter activity. These observations are consistent with the multifaceted nucleic acid binding and chaperoning activities attributed to this protein. Indeed, NCL is most abundant in the nucleolus, but can be found also in cell membranes and, upon stress stimuli, in the nucleoplasm and cytoplasm, to some extent (49,50). Among other functions, it is involved in transcription (50) by specific interactions with sequences that can adopt complex secondary structures (44,51–52). It is widely believed that NCL plays a chaperone role by helping the correct folding of complex nucleic acids structures. Indeed, NCL has been shown to display a marked preference for both endogenous and exogenous G-rich sequences that can fold into G4 (53). It has been recently reported that binding of NCL to the endogenous (GGGGCC)*_n_* hexanucleotide repeat expansion (HRE) in *C9orf72* is responsible for the initiation of molecular cascades that lead to neurodegenerative diseases (19). At the promoter level, binding of NCL to G4 structures augments the basal effect of the folded conformation (45,54–56). One of the best documented example of G4-mediated regulation among G4 promoters (57) is that of c-myc, which shows striking similarities with the G4-mediated regulation of the HIV-1 LTR promoter reported here and previously by us (30). Both cases involve multiple G-tracts that enable folding into alternative G4 conformations; G4 parallel-like topology (58); at least one G_3_N_1_G_3_ motif; binding sites for SP1; silencing effect on promoter activity (59); NCL-binding activity and higher affinity of NCL towards the DNA G4 compared to the RNA G4 counterpart (45). In the case of c-myc, it has been shown that the N-terminal of NCL is dispensable for its G4 binding activity (56). Here, we show that NCL can naturally produce cleaved forms that lack the N-terminal but retain full binding capabilities. These observations demonstrate that cells allow the formation of NCL cleaved species that maintain their G4/nucleic acid binding activity. On the other, they suggest that the HIV-1 virus and human host cells have likely evolved identical mechanisms to control transcription at the DNA promoter level.

The results of our experiments provided valuable insights into the determinants of NCL binding to the various LTR G4-forming structures. The lower binding of NCL to LTR-IV compared to the other G4 structures suggests that the interaction has both conformation- and sequence-dependent characteristics, which in turn implies the fascinating possibility that different G4s in the HIV-1 LTR promoter may exert different functions based on their binding partners. Moreover, the greater affinity demonstrated for DNA than RNA constructs indicates that the interaction is deeply influenced by conformational differences between G4 structures folded by the different types of biopolymers. The facts that the HIV-1 genome consists of RNA, that this G4-forming sequence is also present in the U3 region of the genome (33), and that viral RNA during the first steps of infection is still present in the cell cytoplasm where NCL levels are low, suggest that the specific binding of NCL to the DNA version may represent an essential mechanism for regulating viral transcription. In this direction, it has been shown that NCL is involved in different steps of the HIV-1 life cycle. Inhibition of surface NCL by different cellular and synthetic compounds (60–62) affects cell attachment/entry by the virus (63). In addition, NCL can bind HIV-1 Gag protein to promote viral budding (64), or to enhance Gag release (65). Further, NCL involvement in the viral life cycle has been corroborated also by evidence that HIV infection modifies the protein's cellular distribution (66,67). The activities performed by NCL in other viruses have also been described in a number of recent papers (68–72). For example, binding of NCL to Epstein–Barr virus (EBV) nuclear antigen 1 (EBNA1) modulates viral replication and transcription (73), and virus-induced relocalization of NCL has been observed in some instances (74,75). In this broader context, these reports support our new findings that point toward a significant role played by this protein in HIV-1 replication.

The observation that NCL interaction with LTR G4 silences viral transcription is in apparent contrast with the well-known ability of NCL to interact with histone H1, which induces chromatin decondensation (76) that in turn facilitates the passage of the DNA polymerases (77). However, the opposite effect consisting of NCL-mediated repression of DNA replication has been also reported (49,78–80), and has been attributed to the interaction of NCL with the DNA processing enzyme Replication Protein A (RPA). In addition, NCL has been reported to recruit a DNA helicase that unwinds G4 structures (81). In both cases, NCL binding to cellular proteins is a transient response to stress stimuli. These observations prompt the intriguing possibility that when NCL is redistributed by the cellular stress imposed by HIV infection (66,67), it may be then recruited by the G4 structure of viral promoter to transiently down-regulate HIV transcription and enable the virus to prepare for subsequent efficient transcription, when viral proteins, such as Tat, take over. Alternatively, this interaction may be required as a first switch to viral latency and to recruit proteins that further consolidate latency. The signals that trigger latency are not known at this point and are thus the object of intense studies. However, factors that repress viral transcription at the LTR promoter have been proposed to play a determinant role in latency mechanisms (82,83). Finally, the very specific nature of nucleolin binding indicates that its viral target must be less prone to mutations. This observation makes the viral G4/nucleolin complex into a very appealing target for the development of antiviral strategies that may afford a different mechanism of action, the possibility of targeting viral latency, and a lower probability of incurring into drug resistance. In conclusion, we have shown that the specific binding of the cellular protein NCL to the LTR promoter regulates viral transcription. This result alone paves the way for the investigation of different regulation mechanisms of HIV-1 transcription/latency, which may lead to new possible targets for the design of specific inhibitors.

## Supplementary Material

SUPPLEMENTARY DATA
