# Supplementary material for: Nucleolin stabilizes G-quadruplex structures folded by the LTR promoter and silences HIV-1 viral transcription
Source: Nucleic Acids Res. 2015 Oct 10;43(18):8884–97. doi: 10.1093/nar/gkv897 (PMC4605322; doi:10.1093/nar/gkv897)
Supplement: SUPPLEMENTARY DATA [file supp_43_18_8884__index.html]

Nucleolin stabilizes G-quadruplex structures folded by the LTR promoter and silences HIV-1 viral transcription — SUPPLEMENTARY DATA 

# Nucleolin stabilizes G-quadruplex structures folded by the LTR promoter and silences HIV-1 viral transcription

## SUPPLEMENTARY DATA

- SUPPLEMENTARY DATA
