# Supplementary material for: Nucleolin stabilizes G-quadruplex structures folded by the LTR promoter and silences HIV-1 viral transcription
Source: Nucleic Acids Res. 2015 Oct 10;43(18):8884–97. doi: 10.1093/nar/gkv897 (PMC4605322; doi:10.1093/nar/gkv897)
Supplement: SUPPLEMENTARY DATA [file supp_gkv897_nar-02089-f-2015-File008.pdf]

Table S1. Oligonucleotides used in this study.

Assay	Name	Sequence 5’-3’
EMSA, SPR, DMS footprinting	LTR-II	d(TTTTTGGGGACTTTCCAGGGAGGCGTGGCCTGGGCGGGTTTTT)
	LTR-III	d(TTTTTGGGAGGCGTGGCCTGGGCGGGACTGGGGTTTTT)
	LTR-IV	d(TTTTTGGGCGGGACTGGGGAGTGGTTTTT)
	LTR-II+III+IV	d(TTTTTGGGGACTTTCCAGGGAGGCGTGGCCTGGGCGGGACTGGG GAGTGGTTTTT)
	LTR-III+IV	d(TTTTTGGGAGGCGTGGCCTGGGCGGGACTGGGGAGTGGTTTTT)
	LTR-II+III+IV M4	d(TTTTTGGGGACTTTCCAGGGAGGCGTGGCCTGTGCGGGACTGGG GAGTGGTTTTT)
	LTR-II+III+IV M5	d(TTTTTGGGGACTTTCCAGGGAGGCGTGGCCTGGGCGTGACTGGG GAGTGGTTTTT)
	LTR-II+III+IV M4+5	d(TTTTTGGGGACTTTCCAGGGAGGCGTGGCCTGTGCGTGACTGGG GAGTGGTTTTT)
	LTR-II+III+IV M3”	d(TTTTTGGGGACTTTCCAGGGAGGCGTTGCCTGGGCGGGACTGGG GAGTGGTTTTT)
	Scrambled	d(TTTTTGGAGCGTGTGTGCGCGAGAGCGTGCGCGTGGCGAGCGTG GAGTGGTTTTT)
	c-myc	d(TGGGGAGGGTGGGGAGGGTGGGGAAGG)
	LTR-III+IV	GGGAGGCGUGGCCUGGGCGGGACUGGGGAGUGG
Pull-down	LTR-II+III+IV	d(TTTTTGGGGACTTTCCAGGGAGGCGTGGCCTGGGCGGGACTGGG GAGTGGTTTTT-*BtnTg*)
	LTR-II+III+IV M4+5	d(TTTTTGGGGACTTTCCAGGGAGGCGTGGCCTGTGCGTGACTGGG GAGTGGTTTTT-*BtnTg*)
	Random	d(AAAAACTACTGCACGCTCGCTACGACGACACTGTCGCGCATACAA GCTGCAAAAA-*BtnTg*)
FRET	LTR-II	d(*FAM*-TGGGGACTTTCCAGGGAGGCGTGGCCTGGGCGGGT-*TAMRA*)
	LTR-III	d(*FAM*-TGGGGACTTTCCAGGGAGGCGTGGCCTGGGCGGGACTGGGGT-*TAMRA*)
	LTR-IV	d(*FAM*-TGGGCGGGACTGGGGAGTGGT-*TAMRA*)
	LTR-II+III+IV	d(*FAM*-TGGGGACTTTCCAGGGAGGCGTGGCCTGGGCGGGACTGGGGAGTG GT-*TAMRA*)
	LTR-III+IV	d(*FAM*-TGGGAGGCGTGGCCTGGGCGGGACTGGGGAGTGGT-*TAMRA)*
Taq polymerase	LTR-II+III+IV Taq	d(TTTTTGGGGACTTTCCAGGGAGGCGTGGCCTGGGCGGGACTGGG GAGTGGTTTTTCTGCATATAAGCAGCTGCTTTTTGCC)

stop	LTR-II+III+IV M4+5 Taq	d(TTTTTGGGGACTTTCCAGGGAGGCGTGGCCTGTGCGTGACTGGG GAGTGGTTTTTCTGCATATAAGCAGCTGCTTTTTGCC)

BtnTg: Biotin TEG, *FAM*: 6-carboxyfluorescein,*TAMRA*: 6-carboxy-tetramethylrhodamine

Supplementary Figures

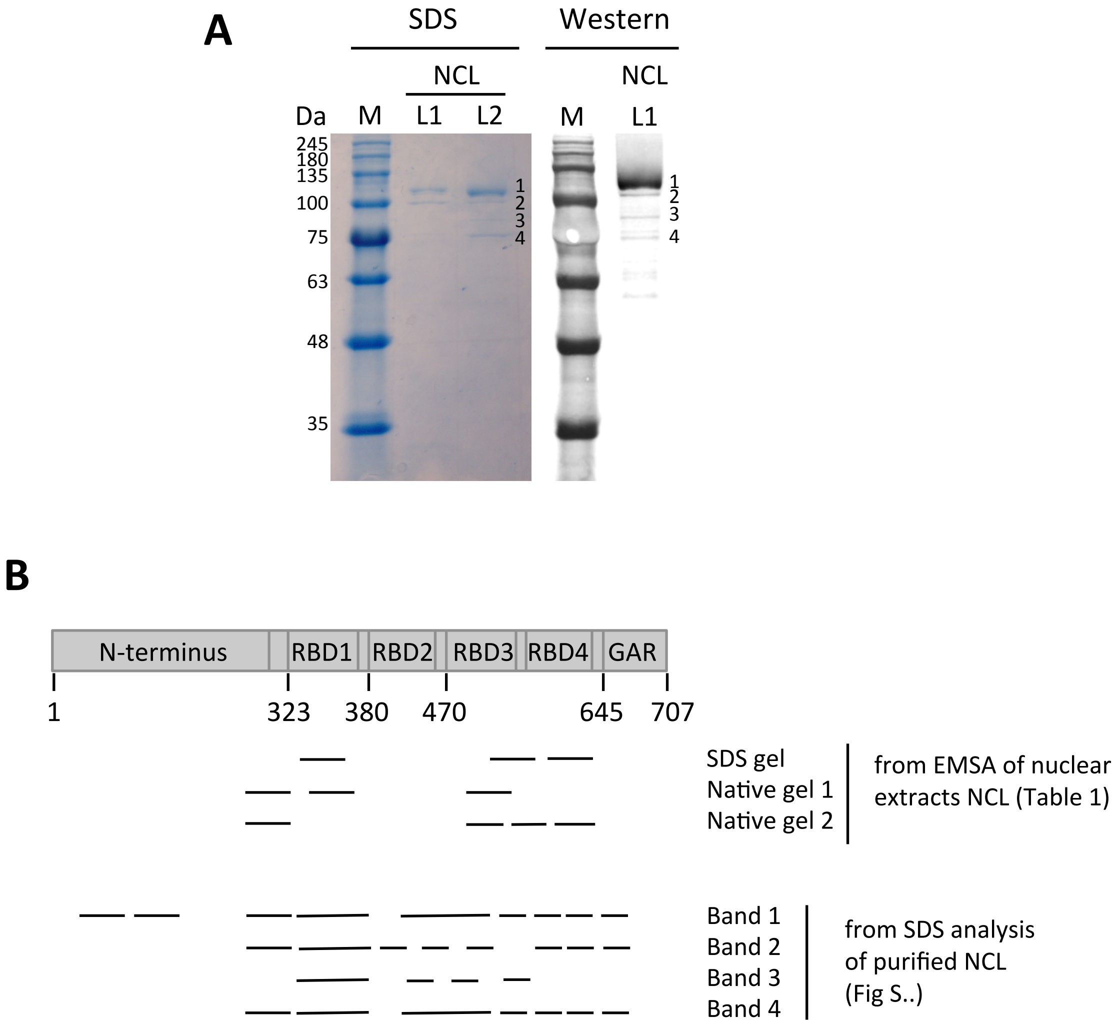

Figure S1. Analysis of the full-length and cleaved form of NCL. A) SDS gel stained with Comassie (SDS, left side) of two different lots (L1 and L2) of purified NCL. Western blot (Western, right side) of one lot (L1) of purified NCL. M indicate protein marker lane. The molecular weights of the protein markers are shown on the left side of the image. B) NCL coverage of peptides obtained by MS analysis. The full-length NCL sequence is shown as a grey rectangle and domains are indicated: RBD stands for RNA-binding domain; GAR stands for glycine/arginine-rich domain. Peptides obtained by MS are shown as horizontal bars; their position corresponds to the position in the NCL protein sequence. The first set of peptides was obtained by analysis of the protein (from nuclear extracts) that was found bound to the wt LTR-II+III+IV G4 in EMSA (see also Table 1). The second set of peptides corresponds to the four bands obtained from the purified NCL run on the Comassie-stained SDS gel.

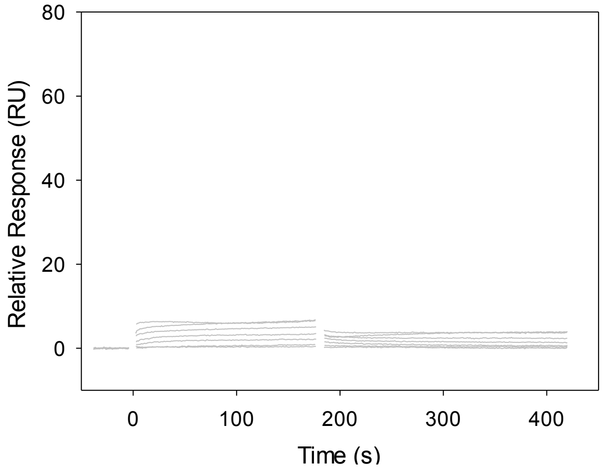

Figure S2. SPR binding analysis of scrambled LTR-II+III+IV to immobilized NCL. Oligonucleotide concentration range was 31.25 nM-2000 nM. Sensograms are shown as gray lines. Fits with binding equations could not be obtained.
